# Operative Vaginal Delivery Compared to Cesarean After Failed Labor: A Population-Based Analysis of Neonatal and Maternal Outcomes

**DOI:** 10.3390/children13040511

**Published:** 2026-04-07

**Authors:** Yvalis Cortes-Rojas, Braxton Forde

**Affiliations:** Department of Obstetrics & Gynecology, University of Cincinnati College of Medicine, Cincinnati, OH 45267, USA

**Keywords:** operative vaginal delivery, vacuum-assisted delivery, forceps-assisted delivery, neonatal morbidity, birth trauma, neonatal sepsis, respiratory distress

## Abstract

**Highlights:**

**What are the main findings?**

- When compared to cesarean in the setting of failed labor, operative vaginal delivery is associated with decreased neonatal respiratory morbidity.
- Operative vaginal delivery is associated with increased laceration-related maternal morbidity compared to cesarean delivery, but decreased hysterectomy and ICU admissions.

**What are the implications of the main findings?**

- US birth registries suggest neonatal benefits of operative vaginal delivery compared to cesarean.
- Continued training on operative vaginal delivery is critical in obstetric training.

**Abstract:**

**Objective:** We sought to compare common neonatal and maternal morbidity outcomes amongst operative vaginal delivery (OVD) versus cesarean delivery performed in the setting of failed attempt at labor. We planned to stratify outcomes by type of OVD (vacuum-assisted vaginal delivery (VAVD) and forceps-assisted vaginal delivery (FAVD)). **Methods:** This was a retrospective cohort study of singleton live births in the United States, using the 2023 National Vital Statistics birth certificate dataset. The primary outcome of interest was the risk of neonatal morbidity, as listed on the birth certificate. The secondary outcome of interest was the risk of maternal morbidity. Neonatal morbidities were planned to be analyzed independently (i.e., risk of NICU admission, need for antibiotics) as well as in aggregate (i.e., the risk of any morbidity occurring). Three groups were planned: FAVD, VAVD, and cesarean in the setting of attempted labor or attempted induction of labor (referent group). Differences in demographic and clinical characteristics were compared and subsequently adjusted for, and odds ratios (aOR) were calculated using multivariable logistic regression. **Results:** Of the 3,605,081 births from 2023, there were 15,384 FAVDs; 83,134 VAVDs; and 325,310 cesareans after failed labor. Neonatal morbidity was lower in FAVD (aOR 0.71, 95% CI 0.66–0.76) and VAVD (aOR 0.57, 95% CI 0.55–0.59) compared to cesarean delivery, with VAVD showing the lowest rates, in particular, the need for assisted ventilation (aOR 0.52 95% CI 0.48–0.57 with VAVD and aOR 0.74 95% CI 0.68–0.81 with FAVD) and NICU admissions aOR 0.66, 95% CI 0.60–0.71 with FAVD and aOR 0.48, 95% CI 0.46–0.51 with VAVD) were reduced with operative vaginal delivery. Antibiotic usage was only reduced in VAVD, not FAVD. Maternal morbidity was highest FAVD; however, this was driven by perineal lacerations. ICU admission, hysterectomy, and ruptured uterus were all higher in cesarean delivery than FAVD or VAVD. **Conclusions:** Operative vaginal delivery, particularly VAVD, is associated with reduced neonatal morbidity compared to cesarean delivery in the setting of labor.

## 1. Introduction

One of the highest risks of maternal morbidity is in the setting of a second-stage cesarean delivery. Even in light of this, cesarean deliveries in the setting of failed labor are on the rise [1,2,3]. This has led to a renewed interest in obstetric training on operative vaginal delivery (OVD). OVD, utilizing either forceps or vacuum, has been consistently shown to reduce maternal complications related to cesarean delivery. When comparing forceps vs. vacuum, there is no significant difference in maternal morbidity outside of perineal lacerations (higher in forceps), and the choice is usually dictated by provider comfort. While method-specific neonatal complications, such as cephalohematoma with vacuum and facial nerve injury with forceps, are well-documented [2,4,5,6,7], broader neonatal outcomes remain insufficiently explored. Contag et al. reported that OVD was associated with an equivalent risk of fetal acidosis or asphyxia when compared to second-stage cesarean delivery; however, this study was limited by its size, having only a few hundred patients. This low N prevented the ability to evaluate rarer neonatal complications [8]. Conversely, vaginal deliveries are well-established as having significant neonatal benefits compared to cesarean deliveries, particularly in regard to respiratory morbidity. It is unknown if OVD provides similar neonatal benefits.

We thus sought to evaluate US birth certificate data to examine the risk of neonatal morbidity with OVD relative to cesarean delivery in the setting of failed labor. Although provider training and institutional norms often influence the choice between VAVD and FAVD, we also sought to examine if there were operative-specific risks or benefits (i.e., comparing vacuum and forceps individually to cesarean deliveries). We hypothesized that OVD would be associated with lower rates of neonatal morbidity compared to cesarean delivery, with particularly lower rates of respiratory morbidity. We also hypothesized that there would be no difference in neonatal morbidity with one type of operative delivery vs. the other (i.e., similar outcomes with both FAVD and VAVD).

## 2. Methods

This was a population-based, retrospective cohort study analyzing all live births in the United States in 2023 using the National Vital Statistics birth certificate dataset. The primary objective was to compare neonatal morbidity among forceps-assisted vaginal deliveries, vacuum-assisted vaginal deliveries, and cesarean deliveries following a failed attempt at labor or failed induction of labor. The secondary objective was to assess maternal morbidity risk between groups. All singleton live births recorded in the 2023 National Vital Statistics System (NVSS) dataset were screened for inclusion. Deliveries were classified based on birth certificate documentation, which specifies the mode of delivery and whether an attempt at labor occurred before a cesarean section. As per the US birth certificate data, a cesarean is considered to occur after labor if labor was attempted and subsequently a cesarean occurred. This cesarean could occur after either an arrest of dilation, arrest of descent, fetal intolerance of labor, or non-reassuring fetal status during the labor process, and the birth certificate coding does not differentiate between these various types of cesareans. These cesareans do NOT include repeat cesarean delivery or cesarean in the setting of failed trial of labor after cesarean (failed TOLAC). This was considered to be a pragmatic referent group as it would include all OVD eligible deliveries, though it must be noted that it also includes OVD ineligible deliveries (such as cesarean for failure to dilate). VAVD and FAVD are coded for in the US birth certificate dataset; however, they are coded as the final route of delivery, not the attempted route. While the birth certificate does list “was a delivery with forceps attempted but was unsuccessful” and “was a delivery with vacuum attempted but was unsuccessful,” for these two specific variables, there are concerns regarding coding inaccuracies in the US Birth Certificate database, and thus, these variables are not included in the analysis. Exclusion criteria included multiple gestations, major congenital anomalies, and missing data on delivery mode or birthweight. Maternal demographic and clinical characteristics extracted included age, parity, race, education level, pre-pregnancy BMI, and chronic medical conditions (chronic hypertension, pregestational diabetes, gestational hypertension, and gestational diabetes). Prenatal factors assessed included the use of assisted reproductive technology (ART), cigarette smoking, lack of prenatal care, and Medicaid insurance status.

We planned to independently analyze VAVD and FAVD compared to the referent group of cesareans in the setting of failed labor to better determine if subtle differences were noted between the types of operative delivery. In both analyses (VAVD vs. cesarean in setting of failed labor and FAVD vs. cesarean in the setting of failed labor), the primary outcome of interest was the composite neonatal morbidity, which was defined as the presence of any of the following: NICU admission, 5 min Apgar score < 7, need for assisted ventilation, neonatal antibiotic administration, and seizure or neurologic dysfunction. Each morbidity was also assessed individually. Similarly, maternal morbidity was evaluated, including ICU admission, blood transfusion, severe perineal tears (third or fourth degree), uterine rupture, and hysterectomy. It should be noted that these outcomes are recorded by the obstetric and neonatal nursing staff on the obstetric record of live birth, and that data is then uploaded to the CDC and NVSS. While the accuracy of all reported variables has been established across health systems, it should be noted that the variables reported are not specific diagnosis codes but rather based upon the reporting on the birth certificate.

Baseline maternal demographics, obstetric characteristics, and neonatal outcomes were compared across delivery groups using chi-square (χ^2^) tests for categorical variables and *t*-tests for continuous variables, as appropriate. Multivariable logistic regression models were used to estimate adjusted odds ratios (aORs) with 95% confidence intervals (CIs) for maternal and neonatal outcomes. Models were planned to undergo adjustment via a backward selection approach, starting with all variables that were significantly different between groups or were biologically plausible to affect the outcome of interest. Variables were then removed in a stepwise fashion until only statistically significant, impactful variables upon the model were included. A backward selection strategy was planned as it can reduce overfitting, as it starts with a larger candidate model and then sequentially removes variables that do not add independent predictive information relative to the sample size. This strategy was used specifically to reduce overfitting, which can occur in multivariate logistical regression models. Statistical significance was defined as *p* < 0.05 or the 95% CI not inclusive of 1. All statistical analyses were conducted using STATA v15.1. (StataCorp, College Station, TX, USA). For transparency of our work, the dataset used, as well as the user handbook for the US Birth Certificate dataset, are available via the following link: https://www.cdc.gov/nchs/data_access/vitalstatsonline.htm, accessed on 4 November 2024. The birth data can be downloaded by year, alongside a user guide for each year.

## 3. Results

There was a total of 3,605,081 live births evaluated for study inclusion, with 15,384 FAVD, 83,134 VAVD, and 325,310 cesareans after failed labor cases. The remainder of cases were either successful vaginal birth, cesarean delivery WITHOUT the attempt at labor, or vaginal birth after cesarean, and those births were excluded from the analysis. Significant differences in maternal demographics and clinical characteristics were observed across the delivery groups (Table 1). The highest proportion of mothers aged 18–34 years was observed in the VAVD group (78.9%), followed by FAVD (78.1%) and CS with failed labor (77.5%). Nulliparity was most frequent in VAVD (22.8%), followed by FAVD (21.9%) and CS failed labor (21.6%). Non-Hispanic white mothers were most prevalent in the FAVD group (55.3%), followed by VAVD (51.2%) and CS failed labor (49.0%). Other demographic factors, such as educational levels, obesity, and chronic medical conditions, also varied significantly across the groups, with CS failed labor exhibiting the highest rates of obesity and chronic conditions such as hypertension and gestational diabetes.

Regarding the primary outcome, neonatal morbidity differed significantly across delivery methods (Table 2), with the lowest neonatal morbidity in the VAVD group. The overall composite neonatal morbidity rate was highest in cesarean with failed labor (20.2%), followed by FAVD (15.5%) and VAVD (13.1%). After backward selection, the final model for both FAVD and VAVD was adjusted for race, BMI, both chronic and gestational hypertension, both pregestational and gestational diabetes, and cigarette use. This was the same model used for all variables, with the exception of seizures or neurologic dysfunction. Due to the very low N, no adjustment was made for these variables to prevent overfitting and preserve the stability of the analysis. After said adjustments for clinical and demographic differences, the adjusted odds ratio (aOR) for composite neonatal morbidity was 0.71 (95% CI 0.66–0.76) for FAVD and 0.57 (95% CI 0.55–0.59) for VAVD, compared to cesarean with failed labor. NICU admissions were also significantly lower in both FAVD (10.6%) and VAVD (8.5%) compared to cesarean with failed labor (14.4%). The aOR for NICU admission was 0.66 (95% CI 0.60–0.71) for FAVD and 0.48 (95% CI 0.46–0.51) for VAVD (Table 2). Similarly, the need for assisted ventilation (aOR 0.52, 95% CI 0.48–0.57 with VAVD and aOR 0.74, 95% CI 0.68–0.81 with FAVD) was reduced with both VAVD and FAVD. Antibiotic usage was only reduced in VAVD (aOR 0.52, 95% CI 0.48–0.57), not FAVD.

The secondary outcomes of maternal morbidity are presented in Table 3. After backwards selection, the final model for both FAVD and VAVD was adjusted for age, race, BMI, chronic hypertension, gestational hypertension, pregestational diabetes, cigarette use, and Medicaid status. This was the same model used for all variables, with the exception of ICU admission, which, due to low N, was only adjusted for BMI and chronic hypertension. Due to the very low N, neither ruptured uterus nor hysterectomy was adjusted for any covariates to prevent overfitting and preserve the stability of the analysis. Maternal morbidity was highest in FAVD. When evaluating individual contributors to maternal morbidity, both FAVD and VAVD morbidity were largely driven by laceration-related morbidity (Table 3). As cesarean delivery carries almost no risk of a perineal laceration, the rate of 3rd or 4th perineal lacerations was significantly higher in OVD, with 13.7% in FAVD (aOR 124.0, 95% CI 100.1–153.7), compared to 5.6% in VAVD (aOR 38.1, 95% CI 31.0–45.0) and 0.06% in CS failed labor. Of note, FAVD carried a similar rate of blood transfusion as cesarean (1.22% vs. 1.35%), while VAVD had significantly lower rates of blood transfusion (0.78%). After adjustment, the odds of blood transfusion were lower both in FAVD and VAVD (aOR 0.57, 95% CI 0.40–0.80 and aOR 0.40, 95% CI 0.33–0.48, for FAVD and VAVD, respectively). Non-laceration, non-transfusion morbidity was lower in both operative groups than in cesareans (aOR 0.42, 95% CI 0.30–0.71 and aOR 0.37, 95% CI 0.28–0.53 for FAVD and VAVD, respectively). (Table 3). ICU admissions were significantly lower in FAVD (0.25%, aOR 0.59, 95% CI 0.37–0.95) and VAVD (0.17%, aOR 0.43, 95% CI 0.33–0.55), than in CS failed labor (0.42%). Overall, while neonatal morbidity appeared to be lowest in the setting of successful VAVD, maternal morbidity was more mixed, with increased lacerations in both FAVD and VAVD, but lower non-laceration related maternal morbidity in OVD, particularly in VAVD.

## 4. Discussion

Our study highlights the possible neonatal benefits of an operative second stage relative to cesarean delivery. Interestingly, while the type of operative delivery is normally dictated by provider comfort (excluding the rare cases of significant prematurity, excluding the ability to use a vacuum), our study found small but significant differences in neonatal morbidity based upon the type of operative delivery. VAVD had the lowest rates of neonatal morbidity, particularly regarding respiratory morbidity and NICU admissions.

Our results are consistent with studies in the literature while highlighting new findings of interest. When compared to spontaneous vaginal delivery, cesarean delivery is associated with increased odds of neonatal respiratory distress and low five-minute Apgar scores, even after adjusting for multiple confounding variables [9]. This persists across both high and low-risk populations, suggesting that the physiological benefits of the birthing process itself may confer early respiratory and adaptive advantages. From a biological perspective, there seems to be some respiratory-specific advantages for a child to go through the birth canal. As early as the 1980s, it was noted that a catecholamine surge occurs with infants who are delivered vaginally, which translates to higher tidal volume and improved respiratory compliance in neonates [10]. Furthermore, infants born via cesarean have higher fluid content in their lungs, likely preventing gas exchange. It is believed that this fluid is both expelled by compression as well as absorbed via catecholamine-driven mechanisms [11]. Our study supports the hypothesis that specific changes occur in the neonatal lungs secondarily to the route of delivery, as there were significantly lower rates of respiratory morbidity with VAVD and FAVD compared to cesarean in the setting of failed labor. Another finding of note in our study is the difference in neonatal sepsis and, thus, antibiotic usage, with significantly lower sepsis and antibiotic use in VAVD than in cesarean for failed labor. While the respiratory benefits of delivery through the birth canal are established, there is less mechanistic data to explain the decreased sepsis and antibiotics usage in neonates delivered via OVD. There is research that supports the differing microbiome development of a neonate delivered via cesarean vs. vaginally, and it is suggested that the early microbiome may shape immune development [12]. Furthermore, gut dysbiosis is linked to neonatal sepsis [13], so OVD may provide subtle microbiome benefits over cesarean delivery. Studies reporting maternal outcomes between operative delivery and cesarean delivery have consistently highlighted a maternal benefit in an operative second stage versus a second stage labor arrest [5,14], findings consistent with the results of our study as well. While lacerations were common in OVD, ICU admission and hysterectomy rates were far lower with OVD than with cesarean. An interesting finding in our study is that both the consistently greater reduction in morbidity with VAVD compared to FAVD, as well as the need for antibiotic usage, was decreased only in VAVD. Given the differences in VAVD and FAVD, further evaluation is warranted to substantiate or refute these findings. This is particularly apropos as FAVD has historically been considered to have higher success rates than VAVD; therefore, one could postulate that the lower morbidity seen with VAVD was from less-complicated or easier-to-deliver infants. However, some research has bucked this historical belief and has noted higher success rates with attempting VAVD than FAVD [15]. Further study of the best type of operative delivery is needed.

Comprehensive evidence indicates that cesarean delivery, while lifesaving, is linked to higher risks of severe acute maternal morbidity (SAMM), abnormal placentation, uterine rupture, and adverse outcomes in subsequent pregnancies [16]. The cumulative maternal risks increase with each repeat cesarean, and with rising rates of hysterectomy and hemorrhage [17], avoidance of cesarean should be attempted if possible. Parallel to this, a critical skill in the avoidance of the primary cesarean section is comfort and skill with OVD. Evidence points to physician experience and hospital volume as key modifiable factors influencing OVD success and safety [14,15,17,18,19], and this was a driving factor in the performance of this study. We wished to see, with the decline in the OVD rate overall, whether there was still a major benefit to the patient and their child with OVD vs. cesarean, and our results seem to support a benefit to OVD. This also highlights, though, the need for continued training, simulation, and mentorship to maintain technical skills amidst declining OVD rates nationally [15]. Hospitals with low OVD volumes had significantly increased rates of maternal and neonatal complications such as obstetric anal sphincter injuries, shoulder dystocia, and brachial plexus injuries, reflecting reduced provider experience and institutional preparedness [18]. These findings underscore the importance of concentrating resources, optimizing provider skill, and implementing quality improvement initiatives to enhance OVD safety, especially in low-volume centers [14,16,17,18,19]. Given the significant maternal and neonatal benefits appreciated in our study, further emphasis on training and continuing education to maximize safety and minimize harm with OVD is warranted.

While there are numerous strengths to this study, a few limitations must be noted. The first critical gap in our understanding lies in the rate of failed VAVD or failed FAVD and its impact on neonatal outcomes. There is existing literature on factors associated with failure of operative vaginal delivery [20,21,22], there remains a lack of evaluation upon failed OVD and neonatal outcomes. While the US certificate of live birth does have variables for “was forceps attempt and unsuccessful” and “was vacuum attempted and was unsuccessful,” this data is not currently available for analysis due to ongoing efforts by NVSS to validate its accuracy. Thus, our analysis focused only upon successful VAVD and successful FAVD when compared to cesarean in the setting of failed labor. There is thus some key selection bias for the patients selected, and we wish not to overstate our findings.

In addition to the lack of failed OVD in our dataset, another key limitation is the retrospective nature of the work, which does not allow for causality to be assessed. Another limitation is the lack of granular data regarding the number of attempts of FAVD or VAVD. Furthermore, there is the limitation of confounding by indication. We do not know why patients were having cesarean deliveries, or even why patients were having OVD. Having an OVD to aid in maternal expulsive efforts has a very different neonatal risk profile than having an OVD for a terminal bradycardia while pushing. Additionally, the risk profile of a second-stage arrest cesarean is very different than a cesarean for failure to dilate. Again, due to this, we do not wish to overstate our findings. Lastly, given that this is database work and not a detailed review of the medical record, while the variables presented have been validated as accurate by the CDC and NVSS, the granularity of detail will always be lacking relative to a chart review. However, a strength of this work is that, in addition to the numerous adjusted maternal and demographic factors, the birthweights and gestational ages were similar in the successful FAVD and VAVD groups, suggesting very similar neonatal patient populations. Additionally, this large number of patients allows for an examination of differences in rarer outcomes, something not possible in smaller prospective studies.

The prior literature and our current study suggest that when OVD is achievable and safe, it not only avoids the short- and long-term risks associated with cesarean delivery but also promotes improved neonatal outcomes. These findings reinforce the importance of promoting vaginal delivery, including OVD, as a viable and often preferable alternative to cesarean section when clinically appropriate.

## 5. Conclusions

In this US birth certificate-based dataset, OVD is associated with reduced neonatal morbidity and NICU admissions compared to cesarean deliveries in the setting of failed labor. VAVD was particularly associated with reduced risk of neonatal morbidity. Prospective work is needed to evaluate these outcome differences further.

## Figures and Tables

**Table 1:** Maternal demographics and clinical characteristics by mode of delivery: comparison of demographic and clinical characteristics across delivery methods, including age, parity, race, BMI, chronic conditions, and prenatal care factors.

Maternal Factors	Forceps N = 15,384	Vacuum N = 83,134	CS Failed Labor N = 325,310	*p*
Age (years)				<0.001
<18	420 (2.7%)	2743 (3.3%)	6663 (2.1%)	
18–34	12,010 (78.1%)	65,604 (78.9%)	251,990 (77.5%)	
>34	2954 (19.2%)	14,787 (17.8%)	66,657 (20.5%)	
Parity				<0.001
1	3367 (21.9%)	18,964 (22.8%)	70,398 (21.6%)	
2	1649 (10.7%)	9083 (10.9%)	34,217 (10.5%)	
3	797 (5.2%)	4401 (5.3%)	17,597 (5.4%)	
4+	958 (6.2%)	4755 (5.7%)	21,119 (6.5%)	
Maternal race				<0.001
Non-Hispanic white	8504 (55.3%)	42,520 (51.2%)	159,429 (49.0%)	
Non-Hispanic black	1602 (10.4%)	9585 (11.5%)	52,565 (16.2%)	
Asian	64 (0.4%)	362 (0.4%)	2167 (0.7%)	
Hispanic	3164 (20.6%)	18,895 (22.7%)	77,282 (23.8%)	
Other	401 (2.6%)	1926 (2.3%)	8423 (2.6%)	
Not listed	164 (1.1%)	819 (0.9%)	2595 (0.8%)	
Maternal education				<0.001
Less than high school	1030 (6.7%)	6079 (7.3%)	20,899 (6.4%)	
High school graduate or GED	3455 (22.5%)	20,113 (24.2%)	81,871 (25.2%)	
Higher than high school	2214 (14.4%)	12,659 (15.2%)	57,443 (17.7%)	
Pre-pregnancy BMI (kg/m^2^)				<0.001
1 Underweight < 18.5	526 (3.4%)	3340 (4.0%)	5473 (1.7%)	
2 Normal 18.5–24.9	6917 (44.9%)	38,231 (46.0%)	96,499 (29.7%)	
3 Overweight 25.0–29.9	4072 (26.5%)	22,131 (26.6%)	86,726 (26.7%)	
4 Obesity I 30.0–34.9	2064 (13.4%)	10,503 (12.6%)	61,611 (18.9%)	
5 Obesity II 35.0–39.9	922 (5.9%)	4523 (5.4%)	36,547 (11.2%)	
6 Extreme Obesity III ≥ 40.0	595 (3.9%)	2764 (3.3%)	32,355 (9.9%)	
9 Unknown or not stated				
Chronic medical condition				
Chronic HTN	410 (2.7%)	1693 (2.0%)	15,887 (4.9%)	<0.001
Pregestational Diabetes	168 (1.1%)	652 (0.8%)	6977 (2.1%)	<0.001
Gestational hypertension	1733 (11.3%)	8290 (9.9%)	54,817 (16.9%)	<0.001
Gestational Diabetes	1091 (7.1%)	6113 (7.4%)	33,353 (10.3%)	<0.001
Prenatal factors				
ART	578 (3.8%)	2174 (2.2%)	10,694 (3.3%)	0.235
Cigarette use	510 (3.3%)	2938 (3.5%)	12,779 (3.9%)	<0.001
No prenatal care	264 (1.7%)	1488 (1.8%)	5047 (1.6%)	<0.001
Medicaid	5058 (32.9%)	30,950 (37.2%)	121,728 (37.4%)	<0.001

**Table 2:** Composite neonatal morbidity by mode of delivery: examination of neonatal outcomes such as morbidity rates, NICU admission, Apgar scores, assisted ventilation, and antibiotic use, showing higher neonatal risks in failed labor CS.

*Composite Neonatal Morbidity*	Forceps N = 15,384	Vacuum N = 83,134	CS Failed Labor Referent Group N = 325,310
Any neonatal morbidity	N = 2391	N = 10,907	N = 65,718
Rate	15.5%	13.1%	20.2%
OR	0.72 (0.67–0.76)	0.61 (0.59–0.62)	
aOR	0.71 (0.66–0.76)	0.57 (0.55–0.59)	
NICU Admission	N = 1644	N = 7066	N = 46,862
Rate	10.6%	8.5%	14.4%
OR	0.71 (0.67–0.75)	0.55 (0.54–0.57)	
aOR	0.66 (0.60–0.71)	0.48 (0.46–0.51)	
5 min Apgar < 7	N = 534	N = 2720	N = 14,186
Rate	3.5%	3.3%	4.3%
OR	0.79 (0.72–0.87)	0.76 (0.72–0.79)	
aOR	0.73 (0.63–0.85)	0.71 (0.66–0.77)	
Assisted ventilation	N = 1399	N = 6537	N = 38,847
Rate	9.1%	7.9%	11.9%
OR	0.74 (0.69–0.78)	0.63 (0.61–0.65)	
aOR	0.74 (0.68–0.81)	0.60 (0.56–0.64)	
Antibiotics	N = 497	N = 1760	N = 10,876
Rate	3.2%	2.1%	
OR	0.97 (0.88–1.1)	0.63 (0.59–0.66)	3.3%
aOR	0.90 (0.79–1.0)	0.52 (0.48–0.57)	
Seizure or neurologic dysfunction	N = 20	N = 70	N = 300
Rate	0.01%	0.08%	0.09%
OR	1.4 (0.89–2.2)	0.91 (0.70–1.2)	

**Table 3:** Composite maternal morbidity by mode of delivery: maternal complications, including perineal lacerations, ICU admission, blood transfusion, and uterine rupture, with forceps delivery posing the highest maternal risks.

*Composite Maternal Morbidity*	Forceps N = 15,384	Vacuum N = 83,134	CS Failed Labor Referent Group N = 325,310
**Any Maternal Morbidity**	N = 2252	N = 5275	N = 5998
Rate	14.6%	6.3%	1.8%
OR	9.1 (8.6–9.6)	3.6 (3.5–3.7)	
aOR	4.7 (4.3–5.1)	1.6 (1.5–1.7)	
3rd or 4th Perinatal Laceration	N = 2104	N = 4651	N = 210
Rate	13.7%	5.6%	0.06%
OR	244.9 (212.3–282.6)	91.5 (79.7–105.1)	
aOR	124. 0 (100.1–153.7)	38.1 (31.0–45.0)	
Blood transfusion	N = 187	N = 651	N = 4379
Rate	1.22%	0.78%	1.35%
OR	0.90 (0.78–1.04)	0.58 (0.53–0.63)	
aOR	0.57 (0.40–0.80)	0.40 (0.33–0.48)	
**Any non-laceration, non-transfusion morbidity**	N = 46	N = 197	N = 2349
Rate	0.30%	0.24%	0.72%
OR	0.42 (0.34–0.52)	0.33 (0.29–0.37)	
aOR	0.42 (0.30–0.71)	0.37 (0.28–0.53)	
ICU Admission	N = 38	N = 139	N = 1371
Rate	0.25%	0.17%	0.42%
OR	0.59 (0.42–0.81)	0.39 (0.33–0.47)	
aOR	0.59 (0.37–0.95)	0.43 (0.33–0.55)	
Hysterectomy	N = 2	N = 35	N = 362
Rate	0.01%	0.04%	0.11%
OR	0.11 (0.03–0.47)	0.38 (0.27–0.53)	
Ruptured Uterus	N = 6	N = 23	N = 616
Rate	0.04%	0.03%	0.19%
OR	0.21 (0.09–0.46)	0.15 (0.09–0.22)	

## Data Availability

All data from this study were obtained from the freely available US Natality files, for which the microdata can be accessed here for download: https://www.cdc.gov/nchs/data_access/Vitalstatsonline.htm, accessed on 4 November 2024.
